# Feasibility of concervative breast surgery and intraoperative radiation therapy for early breast cancer: A single-center, open, non-randomized, prospective pilot study

**DOI:** 10.3892/or.2014.3018

**Published:** 2014-02-11

**Authors:** CARLA CEDOLINI, SERENA BERTOZZI, LUCA SERIAU, AMBROGIO P. LONDERO, SERENA CONCINA, EUGENIA MORETTI, RENATO PADOVANI, ALBERTO PASQUALUCCI, TINO CESCHIA, ANDREA RISALITI

**Affiliations:** 1 Clinic of Surgery, University of Udine, I-33100 Udine, Italy; 2 Clinic of Obstetrics and Gynecology, University of Udine, I-33100 Udine, Italy; 3 Department of Medical Physics, AOU ‘Santa Maria della Misericordia’, I-33100 Udine, Italy; 4 Department of Radiotherapy, AOU ‘Santa Maria della Misericordia’, I-33100 Udine, Italy; 5 Department of Surgical and Biomedical Sciences, University of Perugia, I-06123 Perugia, Italy

**Keywords:** intraoperative radiotherapy, IORT, breast cancer, breast conservative surgery, early breast cancer, overall survival

## Abstract

Intraoperative radiotherapy (IORT) consists of an accelerated, single-dose, partial breast irradiation, performed immediately after breast conservative surgery. In the present study, we report the results of our feasibility protocol study using IORT between 2005 and 2009. We analyzed the data from a single-center, open, non-randomized, prospective pilot study including patients who underwent breast conservative surgery for invasive breast cancer between January 2005 and December 2009 at our Clinic of Surgery. Patients were divided based on IORT performance and stratified by age (≥48 or <48 years). Data were analyzed using R (version 2.15.2), considering a level of significance at P<0.05. Among the 247 eligible patients, 81 accepted the IORT protocol. Intraoperative IORT feasibility was 95.1% (77/81). In 71.4% (55/77) of the cases no postoperative complication was registered. Concerning local recurrence and overall survival, no significant difference was observed between women who underwent the IORT protocol or standard treatment. Among the patients aged <48 years, no local recurrence was noted after IORT protocol, and among women aged ≥48, local recurrences developed later in patients treated with IORT than with standard treatment. IORT represents a feasible and promising technique for the treatment of early breast cancer, with low morbidity, and beneficial aesthetic and oncologic results. Further studies are required in order to extend the inclusion criteria and offer IORT to a larger number of breast cancer patients.

## Introduction

Intraoperative radiotherapy (IORT) is a form of accelerated partial breast irradiation (PBI), which consists of the administration of an accelarated single-dose irradation to the residual breast surrounding the resection bed, immediately after breast conservative surgery during the same anaesthesiologic period. It aims to decrease the overall treatment time and to reduce the amount of normal tissue treated that is not close to a defined visible partial breast target (1).

Moreover, such an approach could theoretically increase the rate at which breast conservative surgery is chosen as a treatment option. It also offers the potential advantages of reduced treatment-related toxicities, provides a logistically faster, convenient, and more accessible method for breast conservative treatment, and potentially improves the overall quality of life for early stage breast cancer patients (2).

IORT may represent an exclusive radiation therapy or an intraoperative boost followed by a shorter external beam radiation therapy (EBRT). The rational for this procedure arose by the observation that loco-regional recurrences after breast conservative surgery mostly develop on the breast parenchyma near the resection bed (3) within 2–3 years after surgery (3–5), whereas after this time interval and outside of the resection bed, eventual neoplastic foci are thought to be new breast cancers rather than recurrences.

Concerning patient outcome, it was demonstrated that local control of disease is comparable in cases of PBI and conventional EBRT, with a recurrence rate following 5 years of follow-up of ~2% (6).

The objective of the present study was to analyze IORT feasibility, complications and outcome in our setting.

## Materials and methods

### Study design and endpoints

The present study was designed as a single-center, open, non-randomized, prospective pilot study. The primary considered endpoint of our study was IORT feasibility. Secondary endpoints were postoperative complications, local recurrence rate and overall survival. The study was initiated after approval by the local institutional ethics committee. The study design was approved by the ethics committee. IORT was offered to all patients undergoing breast conservation for invasive ductal carcinoma and complied with all of the inclusion criteria. Patients who, despite meeting all of the inclusion criteria, did not accept the IORT procedure were considered as the control group. In addition, the control group also consisted of all patients who, despite compliance with the inclusion criteria, did not receive IORT due to technical problems. Furthermore, the IORT procedure protocol differed for women <48 or ≥48 years of age as explained in the IORT protocol treatment section.

### Data collection

We prospectively collected the data of IORT candidate patients who underwent breast conservative surgery for invasive ductal carcinoma of the breast between January 2005 and December 2009. The data included patient age at diagnosis, body mass index (BMI), familial history of breast cancer, fertility status, eventual use of estroprogestinic therapies, TNM classification and stage, nuclear grading, Mib1/Ki-67 proliferation index, estrogen receptor (ER) expression, progesterone receptor (PR) expression, Her2/neu expression, eventual involvement of extra-axillary lymph nodes (internal mammary chain or subclavear ones), tumor multifocality (>1 neoplastic foci within the same quadrant) or multicentricity (>1 neoplastic foci in different quadrants), extensive intraductal component (>25%), perivascular invasion, peritumoral inflammation, perinodal extracapsular invasion or blanched lymph nodes. Moreover, the therapeutic management assessed, including conservative vs. radical, breast and axillary surgery, eventual neoadjuvant therapies, EBRT, and adjuvant endocrine therapy or chemotherapy.

### Inclusion and exclusion criteria

Inclusion criteria for IORT included the following: age between 18 and 80 years, informed consent, unifocal and unicentric disease, ductal invasive histotype, tumor size <3 cm, lymph node status N0 or N1mi, distance of the nearest resection margin from the tumor >5 mm by intraoperative histological examination, acceptable breast volume after quadrantectomy. Exclusion criteria included the following: presence of systemic metastasis, coexistence of a second primary tumor, history of sclerodermia or systemic lupus eritematoides, pregnancy, tumor localization near the areola in the central quadrant and perivascular invasion.

### Preoperative patient assessment

Patients indicated for IORT preoperatively underwent mammography and breast ultrasound, with the placement of a wire hook in case of non-palpable lesions, breast MRI in order to exclude multifocality or multicentricity (7), chest radiography, abdominal ultrasound and total-body bone scintigraphy in order to exclude systemic metastasis. Assessment by a radiotherapy specialist and by a plastic surgeon was scheduled to plan breast remodeling after quadrantectomy and IORT.

### Breast conservation surgery

Quadrantectomy was performed by monopolar cutting and coagulation. Then, the specimen underwent intraoperative radiologic control and frozen-section histological examination in order to confirm tumor size and to measure the tumor distance form the surgical resection margins. In case of tumor distance <5 mm from the surgical resection margins, we performed a margin enlargement before IORT. In all cases, two metallic clips were placed on the resection bed and two on the residual mammary gland surrounding the quadrantectomy, to mark the site for the radiological follow-up.

### Sentinel lymph node biopsy

Intraoperative detection of the sentinel node was guided by a hand-held gamma probe after the subdermal injection of 2.5 ml of human serum albumin macroaggregate (particle size 0.1–0.8 mm) labeled with 74 mBq 99m-technetium 3–24 h before surgery. Every excised sentinel node was then submitted to intraoperative, histological examination of 20 hematoxylin and eosin-stained, 0.15 mm, frozen sections, as well as immunhistochemical evaluation of three random sections to search for an eventual positivity for cytokeratins. Secondary CALND was performed using the same anaesthesiologic period in the case of sentinel node positivity for macrometastases or micrometastases (8,9), while ITCs did not receive further interventions (9,10).

### IORT treatment protocol

Patients satisfying all inclusion criteria were prepared for IORT as follows. After the tumor removal, the residual mammary gland was mobilized from the pectoralis major muscle, on which a dedicated disc of lead and aluminium of various diameters, according to the size of the target, was placed to minimize the radiation delivered to the chest wall and to guarantee the delivery of the full radiation dose to the target. Thereafter, the gland was also mobilized superficially from the skin, and then the residual gland tissue was stitched together directly on the protective disc.

The gland parenchyma thickness was measured using a needle at least in three points, along a hypothetical triangle into the portion of the breast target, to define the correct energy of the electron beam and to calculate the radiation dose. The target area was chosen together by the surgeon and the radiotherapy specialist based on tumor size and location.

A sterile cylindrical applicator was then introduced through the skin incision directly to the breast tissue. The skin was held far from the applicator by a skin retractor system, and a wet gauze was placed between the applicator edge and the displaced skin in order to better protect this from radiation. Finally, a polymethyl methacrylate collimator was docket onto the upper end cylinder attached to the mobile linear accelerator.

The mobile linear accelerator LIAC (Info & Tech, Udine, Italy), which has a robotic arm and measures 230 cm in length, 80 cm in width and 185 cm in height was used. The LIAC has 4 energy settings that can be set to 4, 6, 8 and 10 MeV.

After the surgical room was evacuated, the patients aged ≥48 years were administered an intraoperative exclusive radiation treatment with a single dose of 21 Gy prescribed at 90% isodose curve. The patients aged <48 years were administered an intraoperative boost dose of 10 Gy at 100% isodose, followed by adjuvant EBRT with X photons with a variable dose of 46 Gy, for the first time and then with 50 Gy. Accurate hemostasis control, breast remodeling and wound closure were performed by a plastic surgeon.

### Standard radiation treatment

Patients excluded from the IORT protocol treatment were submitted to standard adjuvant radiotherapy, which consisted of the administration of whole breast radiation with a radiating dose from 46 Gy in 23 fraction to 50 Gy in 25 fraction, as mentioned above. The patients, requiring adjuvant chemotherapy, started the radiation therapy at the end of the chemotherapy protocol. The radiotherapy started within 8 weeks after surgery or 4 weeks from the end of the adjuvant chemotherapy. The CT radiation therapy simulation was generally performed 4 weeks following surgery or, in the case of previous adjuvant chemotherapy, during the last chemotherapy cycle. All patients were treated using a linear accelerator, using 6 or 10 MV, and received a tangent pair radiotherapy to the whole breast. The dose was calculated by ICRU prescription when the dose was 50 Gy.

### Follow-up

Patient follow-up consisted of yearly mammography and breast ultrasound until the 5th year after intervention, and regular controls by a plastic surgeon and a radiotherapy specialist.

### Statistical analysis

Data were analyzed using R (version 2.15.2) and results were considered statistically significant at P<0.05. Univariate analysis was performed by one-way ANOVA or t-test in the case of continuous variables; Chi-square test or Fisher’s exact test in the case of categorical variables. Data are presented as proportions with relative 95% confidence intervals where appropriate. Cumulative events and Kaplan-Meier curves were drawn to compare local recurrence and overall survival in the two groups.

## Results

### Description of the study population (Fig 1)

Among the 1,214 women treated for breast cancer during the study period, 443 were eligible for breast conservative surgery. Among these, 196 patients did not satisfy preoperative or intraoperative inclusion criteria. In particular, 49 ductal invasive cancers presented intraoperatively pathologic exclusion criteria (pN>N1mic); among these, in 30 cases, IORT was not available, 8 patients did not gave their consent, and in 11 cases, despite patient consent, IORT was not performed due to intraoperative nodal upstaging.

Therfore, 247 women satisfied all preoperative and intraoperative inclusion criteria and were eligible for the present study. In 111 cases the procedure was not performed due to technical problems independent of the operators, as IORT was not available for several months and on several occasions. IORT protocol was proposed to 153 eligible women, among which 81 accepted the IORT performance and 55 refused to provide informed consent for the IORT procedure. In 4 cases, despite that all inclusion criteria were met, IORT was not feasible due to insufficient residual breast tissue after quadrantectomy. Finally, 77 eligible women were treated according to the IORT protocol (4 women aged <48 years and 72 aged ≥48 years), whereas 170 eligible patients underwent standard treatments.

Table I describes the characteristics of our study population. The mean age at surgery was 59.78±9.98 years and 85.4% of women (211/247) were in post-menopausal status. The majority of women were aged ≥48 years, and their characteristics are documented in Tables II and III. Among them, 125 received standard EBRT, 73 IORT and 18 did not receive any breast irradiation because of individual comorbidity-related risks or refusal of consent due to personal reasons.

As shown in Table II, major differences were found between patients treated by EBRT or IORT and patients excluded from radiotherapy. In fact, there was a significantly increased prevalence of secondary mastectomies, due to the shorter tumor distance from the surgical resection margins and worse tumor characteristics. Among the patients who received IORT, two women underwent a secondary mastectomy and three underwent further margin widening due to tumor cells close to or infiltrating the surgical margin. Finally, a salvage mastectomy was offered after 5 years of follow-up to one woman who was diagnosed with local recurrence after IORT.

In Tables IV and V, the characteristics of the patients aged <48 years are documented.

### Treatment feasibility

During the study period, 81 eligible patients accepted the IORT protocol, while the treatment was feasible only for 77 (95.1%). In fact, despite the fulfillment of all preoperative and intraoperative inclusion criteria and IORT availability, in 4 cases (4.9%) IORT was not intraoperatively feasible due to a technical impossibility of irradiating residual breast tissue. In particular, IORT was not feasible due to insufficient residual breast tissue in the case of small-sized breasts and marginal localization of the lesion.

### Postoperative complications

In 71.4% (55/77) of the cases, no postoperative complication was noted. Twelve women suffered from wound dehiscence or fat necrosis, which in most cases was treated with cycles of outpatient medications without affecting the aesthetic result, while 1 patient required reoperation to perform wound toilet with complete resolution of sequelae 4 months after IORT. Also 6 cases of seroma were reported, that were easily drained under ultrasound guidance during the postoperative follow-up. Among the 170 control patients treated with conservative surgery, the prevalence of postoperative wound dehiscence or fat necrosis was 14.1% (24/170), which was significantly lower than that of the IORT patients (P<0.05).

### Local recurrence and overall survival

**Figure 2:**
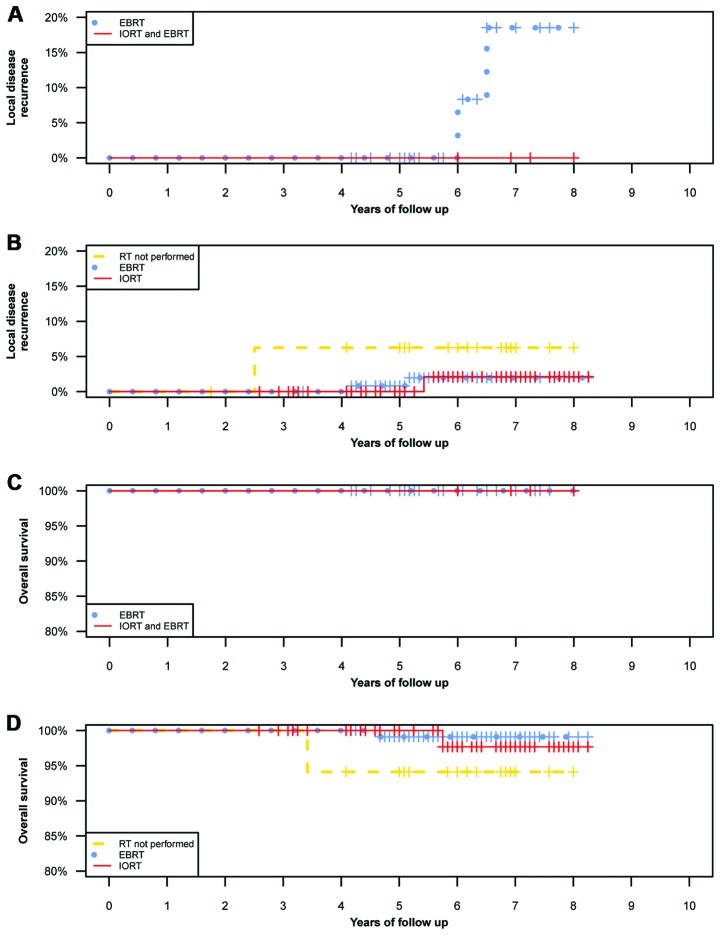
Cumulative local recurrences: (A) patients <48 years of age (P=0.414); (B) patients ≥48 years of age (P=0.442). Overall survival: (C) patients <48 years of age (P=1.000); (D) patients ≥48 years of age (P=0.293).

Fig. 2 shows respectively the local disease recurrence and the overall survival rates. Fig. 2A concerns local disease recurrences among women aged <48 years. Patients treated with combined IORT and EBRT had no recurrences, while the group treated only with EBRT presented a cumulative local recurrence rate of 8.3% (95% CI, 0–22.7) at 6 years of follow-up. However, this difference was not statistically significant. The differences shown in Fig. 2B were not statistically significant, even when local recurrences developed earlier in patients not treated with IORT than in patients treated with the IORT protocol. The observed differences in overall survival results were not statistically significant (Fig. 2C and D).

## Discussion

Among the 1,214 women treated for breast cancer during the study period, 247 were eligible, 81 accepted to receive IORT, and 77 did not receive the IORT treatment (4 women aged <48 and 72 aged ≥48). Intraoperative IORT feasibility was 95.1%. The postoperative complication rate of women undergoing IORT was 28.6%, and thus was significantly higher than that of the controls. Concerning local recurrence and overall survival, no significant difference was observed between women who underwent the IORT protocol or standard treatment. Among the patients aged <48 years, no local recurrence was noted after IORT protocol, and among women aged ≥48 years, local recurrences developed later in patients treated with IORT than in patients treated with EBRT.

If we considered the primary endpoint of the present study, overall IORT feasibility was 95.1%. In four women, IORT unfeasibility was determined by insufficient residual breast tissue after quadrantectomy. Actually, it is extremely difficult to compare our data with those of the literature because of the different interpretations of ‘feasibility’ by other authors (11). In fact, in our opinion, feasibility of a procedure was defined by the technical possibility to perform it in patients who complied with all inclusion criteria required. Therefore, informed consent refusal or intraoperative pT and pN upstaging should not affect IORT feasibility, as they represent absolute exclusion criteria. Moreover, accelerator unavailability should not impact the feasibility of the IORT procedure, which would have been performed on the same patient if the accelerator had been available.

Concerning the procedure toxicity, in 71.4% of cases no postoperative complication was noted, and most local complications were treated with simple outpatient medications and superior aesthetic results were achieved. Although it is difficult to compare EBRT complications with those of IORT, as these are different techniques, and although it was not an objective of the present study, we did observe that IORT usually prevented complications most frequently noted in EBRT. In particular, no sub-/cutaneous lesion (actinic dermatitis, lymphangitis, scar retraction) or chest complication (pulmonary fibrosis, nervous lesions) was observed, and no women reported breast pain after the procedure. IORT was found to be associated with a greater prevalence of fat necrosis than standard EBRT (12,13), particularly in older women who consequently had a higher intra-mammary fat component. In our population, postoperative prevalence of wound dehiscence and fat necrosis was significantly higher in patients who underwent IORT than in patients submitted to the standard treatment, and 6 of our 11 cases of fat necrosis occurred in patients with an age older than that of the mean population.

The cumulative local recurrence rate represents important outcome of IORT. The local reccurence of the patients treated with the IORT protocol was 0% in women aged <48 years and 2% in those aged ≥48 years, and was comparable with most studies of IORT (11,14–21). Moreover, taking into consideration the distance between the previous tumor site and recurrence, our single case of recurrence was very suggestive of a second, ipsilateral, primary tumor. However, local recurrence did not impact patient overall survival, and most authors report 5-year overall survival rates of 100% (11,22). In our population, 76 patients who underwent IORT were alive (98.7%), while one woman developed distant metastasis and died during the follow-up.

Along with the encouraging local control of disease and the satisfying aesthetic results, another important aspect that makes IORT more attractive concerns its economic and logistic advantages. Many resources are necessary for conventional EBRT, which requires about 6–9 personnel hours to plan the treatment, approximately 30 h for patients to receive the treatment, a TC simulator and a LINAC accelerator. Many professional staff must be employed in the execution of the therapy, including two radiotherapy technicians for every fraction for 25 treatments, a physician and one medical physician, while the IORT procedure employs only a radiotherapy technician, a radiation oncologist and a physician. For these and other reasons, the cost of EBRT is very high and is more expensive than that of IORT, for which the main cost is the LINAC.

Finally, it was demonstrated that breast conservative surgery depends on the age of the patient, on the geographic region of the patient, and on the distance from radiotherapy centers (2,23–25). Age is also the major influencing factor for patient compliance to EBRT after breast conservative surgery, which results in ~18% of women aged <50 up to 37% in patients aged >70 years (2). Therefore, IORT may be a feasible solution for elderly women who live in remote rural regions far from hospitals.

The weakness of the present study was the limited number of patients who received IORT. On the other hand, its strength is the long follow-up time and the high reliability of the procedure, which was performed by the same team during the entire study period in a standardized manner.

In conclusion, IORT represents a promising technique for the treatment of early breast cancer, with low morbidity and beneficial aesthetic and oncologic result. It shortens the radiation course to one single session, with an evident economic and logistic benefit. Further studies are required in order to extend the inclusion criteria and allow a larger number of breast cancer patients to receive IORT.

## Figures and Tables

**Figure 1:**
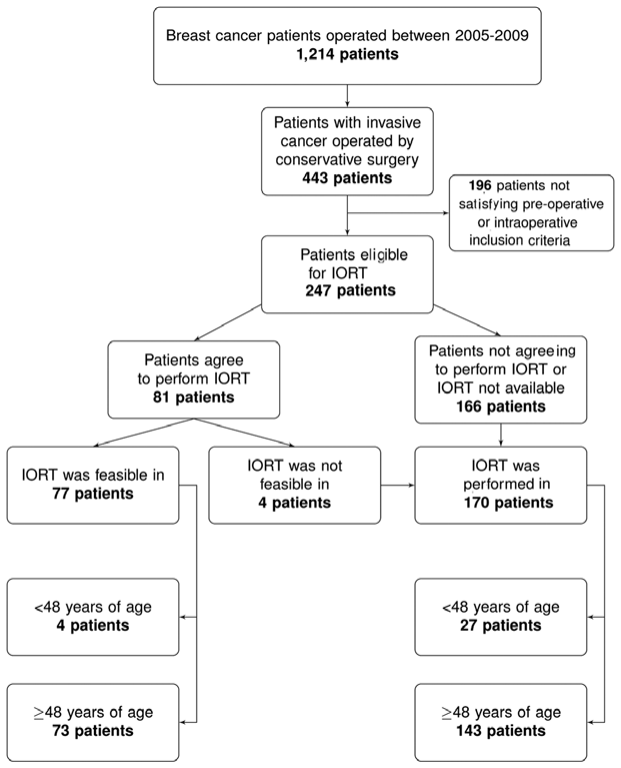
Study flow-chart.

**Table I:** Characteristics of the study population.

Characteristics	Data values
Age (years), mean ± SD	59.78±9.98
BMI (kg/m^2^), mean ± SD	26.41±4.84
Months of follow-up, mean ± SD	71.65±16.01
Tobacco smoking	4.9% (12/247)
Familial history of breast cancer	8.1% (20/247)
Estroprogestinic use	4% (10/247)
Menopausal status	85.4% (211/247)
Diagnosis by screening	30.1% (65/216)
Treatment by patient age	
Age <48 years	
RT	87.1% (27/31)
IORT and RT	12.9% (4/31)
Age ≥48 years	
No RT	8.3% (18/216)
RT	57.9% (125/216)
IORT	33.8% (73/216)
Rescue mastectomy	8.1% (20/247)

BMI, body mass index; RT radiotherapy; IORT, intraoperative radiotherapy. Data values are in % (n/total) unless specified otherwise.

**Table II:** Description of the patients ≥48 years of age treated or not with IORT.

	RT not performed (n=18)	EBRT (n=125)	IORT (n=73)	P-value
Age (years) mean ± SD	63.67±8.88	61.41±8.09	63.32±7.43	0.196
BMI (kg/m^2^) mean±SD	27.47±3.96	26.64±5.04	27.14±4.54	0.700
Months follow-up mean±SD	71.65±14.5	72.29±14.42	69.46±19.2	0.497
Minor margin distance (mm) mean ± SD	5.49±5.56	6.86±5.1	6.29±4.22	0.518
Tobacco smoking, % (n/total)	13.3% (2/15)	1.7% (2/115)	7.2% (5/69)	0.051
Familial history of breast cancer, % (n/total)	100% (3/3)	42.1% (8/19)	16.7% (3/18)	<0.05
Estroprogestinic usage, % (n/total)	0% (0/18)	3.2% (4/125)	5.5% (4/73)	0.490
Menopausal status, % (n/total)	94.1% (16/17)	97.6% (122/125)	100% (73/73)	0.213
Definitive axilla intervention, % (n/total)				
CALND	22.2% (4/18)	8.8% (11/125)	4.1% (3/73)	<0.05
SLNB	77.8% (14/18)	91.2% (114/125)	95.9% (70/73)	<0.05
Second breast surgery, % (n/total)				
Margin widening	5.6% (1/18)	16.8% (21/125)	4.1% (3/73)	<0.05
Mastectomy	72.2% (13/18)	0% (0/125)	2.7% (2/73)	<0.05
Other adjuvant therapies, % (n/total)				
Chemotherapy	33.3% (6/18)	19.2% (24/125)	12.5% (9/72)	0.109
Hormone therapy	66.7% (12/18)	88% (110/125)	84.7% (61/72)	0.059

**Table III:** Tumor characteristics, TNM stage and outcome of patients ≥48 years of age.

	RT not performed (n=18)	EBRT (n=125)	IORT (n=73)	P-value
Tumor characteristics, % (n/total)				
Ki-67/Mib-1 >20	60% (9/15)	21.7% (23/106)	15% (9/60)	<0.05
Comedo-like necrosis	16.7% (3/18)	10.4% (13/125)	2.7% (2/73)	0.070
Extensive intraductal component	55.6% (10/18)	36.8% (46/125)	23.3% (17/73)	<0.05
Molecular subtype, % (n/total)				
Basal-like	11.1% (2/18)	8% (10/125)	5.5% (4/73)	0.664
HER enriched	16.7% (3/18)	1.6% (2/125)	0% (0/73)	<0.05
Luminal A	33.3% (6/18)	60% (75/125)	68.5% (50/73)	<0.05
Luminal B	33.3% (6/18)	28% (35/125)	24.7% (18/73)	0.734
Luminal HER	5.6% (1/18)	2.4% (3/125)	1.4% (1/73)	0.569
Lymph node features, % (n/total)				
Isolated tumor cells	0% (0/18)	1.6% (2/125)	1.4% (1/73)	0.863
Micrometastasis	16.7% (3/18)	4.8% (6/125)	4.1% (3/73)	0.097
TNM classification, % (n/total)				
T1a	11.1% (2/18)	11.2% (14/125)	8.2% (6/73)	0.792
T1b	22.2% (4/18)	34.4% (43/125)	49.3% (36/73)	<0.05
T1c	66.7% (12/18)	54.4% (68/125)	42.5% (31/73)	0.107
N0	83.3% (15/18)	96% (120/125)	95.9% (70/73)	0.066
N1	16.7% (3/18)	4% (5/125)	4.1% (3/73)	0.066
TNM stage, % (n/total)				
I	77.8% (14/18)	88% (110/125)	94.5% (69/73)	0.090
II	22.2% (4/18)	12% (15/125)	5.5% (4/73)	0.090
Grade, % (n/total)				
G1	22.2% (4/18)	36% (45/125)	43.1% (31/72)	0.239
G2	27.8% (5/18)	45.6% (57/125)	41.7% (30/72)	0.350
G3	50% (9/18)	18.4% (23/125)	15.3% (11/72)	<0.05
Local recurrence (N)	(1)	(2)	(1)	
At 1 year	0.0% (0.0-0.0%)	0.8% (0.0–2.4%)	0.8% (0.0–2.4%)	
At 3 years	6.2% (0.0–17.4%)	0.8% (0.0–2.4%)	0.8% (0.0–2.4%)	
At 5 years	6.2% (0.0–17.4%)	0.8% (0.0–2.4%)	0.8% (0.0–2.4%)	
At 6 years	6.2% (0.0–17.4%)	2.0% (0.0–4.6%)	2.0% (0.0–6.2%)	0.442

RT, radiotherapy; EBRT, external beam radiation therapy; IORT, intraoperative radiotherapy; BMI, body mass index.

**Table IV:** Description of the patients <48 years of age treated or not with IORT.

	Only EBRT (n=27)	IORT and EBRT (n=4)	P-value
Age (years) mean ± SD	43.48±2.99	36.75±3.4	<0.05
BMI (kg/m^2^) mean ± SD	23.12±4.23	23.5±1	0.702
Months of follow-up, mean ± SD	72.67±14.89	84.5±9.95	0.092
Minor margin distance (mm) mean ± SD	4.54±5.68	8.25±4.35	0.194
Tobacco smoking, % (n/total)	12% (3/25)	0% (0/4)	0.464
Familial history of breast cancer, % (n/total)	75% (6/8)	0% (0/4)	<0.05
Estroprogestinic usage, % (n/total)	3.7% (1/27)	0% (0/4)	0.696
Definitive axilla intervention, % (n/total)			
CALND, complete axillary lymph node dissection	11.1% (3/27)	0% (0/4)	0.483
SLNB, sentinel lymph node biopsy	88.9% (24/27)	100% (4/4)	0.483
Second breast surgery, % (n/total)			
Margin widening	14.8% (4/27)	0% (0/4)	0.409
Other adjuvant therapies, % (n/total)			
Chemotherapy	30.8% (8/26)	100% (4/4)	<0.05
Hormone therapy	88.5% (23/26)	75% (3/4)	0.461

**Table V:** Tumor characteristic and TNM stage of patients <48 years of age.

	Only EBRT (n=27)	IORT and EBRT (n=4)	P-value
Tumor characteristics, % (n/total)			
Ki-67/Mib-1 >20	21.7% (5/23)	100% (4/4)	<0.05
Comedo-like necrosis	18.5% (5/27)	0% (0/4)	0.347
Extensive intraductal component	44.4% (12/27)	25% (1/4)	0.462
Molecular subtype, % (n/total)			
HER enriched	7.4% (2/27)	0% (0/4)	0.574
Luminal A	66.7% (18/27)	25% (1/4)	0.110
Luminal B	25.9% (7/27)	0% (0/4)	0.247
Luminal HER	0% (0/27)	75% (3/4)	<0.05
Lymph node features, % (n/total)			
Isolated tumor cells	3.7% (1/27)	0% (0/4)	0.696
Micrometastasis	7.4% (2/27)	0% (0/4)	0.574
TNM classification, % (n/total)			
T1a	14.8% (4/27)	0% (0/4)	0.409
T1b	37% (10/27)	0% (0/4)	0.139
T1c	48.1% (13/27)	100% (4/4)	0.052
N0	92.6% (25/27)	100% (4/4)	0.574
N1	7.4% (2/27)	0% (0/4)	0.574
TNM stage, % (n/total)			
I	92.6% (25/27)	100% (4/4)	0.574
II	7.4% (2/27)	0% (0/4)	0.574
Grade, % (n/total)			
G1	33.3% (9/27)	0% (0/4)	0.170
G2	48.1% (13/27)	100% (4/4)	0.052
G3	18.5% (5/27)	0% (0/4)	0.347

RT, radiotherapy; EBRT, external beam radiation therapy; IORT, intraoperative radiotherapy; BMI, body mass index; CALND, complete axillary lymph node dissection; SLNB, sentinel lymph node biopsy.
